# Culturomics- and metagenomics-based insights into the soil microbiome preservation and application for sustainable agriculture

**DOI:** 10.3389/fmicb.2024.1473666

**Published:** 2024-10-24

**Authors:** Elisa Clagnan, Manuela Costanzo, Andrea Visca, Luciana Di Gregorio, Silvia Tabacchioni, Eleonora Colantoni, Filippo Sevi, Federico Sbarra, Arianna Bindo, Lorenzo Nolfi, Rosaria Alessandra Magarelli, Mario Trupo, Alfredo Ambrico, Annamaria Bevivino

**Affiliations:** ^1^Sustainable AgriFood Systems Division, Department for Sustainability, Casaccia Research Center, ENEA, Italian National Agency for New Technologies, Energy and Sustainable Economic Development, Rome, Italy; ^2^Gruppo Ricicla Labs, Department of Agricultural and Environmental Sciences-Production, Landscape, Agroenergy (DiSAA), University of Milan, Milan, Italy; ^3^Department of Life Sciences and System Biology (DBIOS), University of Turin, Turin, Italy; ^4^Department of Agricultural, Forest and Food Sciences (DISAFA), University of Turin, Turin, Italy; ^5^Department of Agriculture and Forest Sciences, University of Tuscia, Viterbo, Italy; ^6^Sustainable AgriFood Systems Division, Department for Sustainability, Trisaia Research Center, ENEA, Italian National Agency for New Technologies, Energy and Sustainable Economic Development, Rome, Italy

**Keywords:** microbiome-based solutions, soil health, microbiome preservation, SynComs, NatComs, omics approaches, microbiome application, sustainable agriculture

## Abstract

Soil health is crucial for global food production in the context of an ever-growing global population. Microbiomes, a combination of microorganisms and their activities, play a pivotal role by biodegrading contaminants, maintaining soil structure, controlling nutrients’ cycles, and regulating the plant responses to biotic and abiotic stresses. Microbiome-based solutions along the soil-plant continuum, and their scaling up from laboratory experiments to field applications, hold promise for enhancing agricultural sustainability by harnessing the power of microbial consortia. Synthetic microbial communities, i.e., selected microbial consortia, are designed to perform specific functions. In contrast, natural communities leverage indigenous microbial populations that are adapted to local soil conditions, promoting ecosystem resilience, and reducing reliance on external inputs. The identification of microbial indicators requires a holistic approach. It is fundamental for current understanding the soil health status and for providing a comprehensive assessment of sustainable land management practices and conservation efforts. Recent advancements in molecular technologies, such as high-throughput sequencing, revealed the incredible diversity of soil microbiomes. On one hand, metagenomic sequencing allows the characterization of the entire genetic composition of soil microbiomes, and the examination of their functional potential and ecological roles; on the other hand, culturomics-based approaches and metabolic fingerprinting offer complementary information by providing snapshots of microbial diversity and metabolic activities both in and *ex-situ*. Long-term storage and cryopreservation of mixed culture and whole microbiome are crucial to maintain the originality of the sample in microbiome biobanking and for the development and application of microbiome-based innovation. This review aims to elucidate the available approaches to characterize diversity, function, and resilience of soil microbial communities and to develop microbiome-based solutions that can pave the way for harnessing nature’s untapped resources to cultivate crops in healthy soils, to enhance plant resilience to abiotic and biotic stresses, and to shape thriving ecosystems unlocking the potential of soil microbiomes is key to sustainable agriculture. Improving management practices by incorporating beneficial microbial consortia, and promoting resilience to climate change by facilitating adaptive strategies with respect to environmental conditions are the global challenges of the future to address the issues of climate change, land degradation and food security.

## 1 Introduction

The increased demand for food due to the world’s growing population has for years caused intensified pressure on natural resources. The introduction of new crops with high-yield genetically improved and the use of new technologies in agriculture, such as chemical fertilizers, herbicides and synthetic pesticides, have led to considerable increase in production and productivity, but at a high environmental cost that is no longer sustainable today. There is a general consensus on the need to define and adopt more sustainable and environmentally friendly agricultural alternatives (Reidsma et al., 2023). Increasing food production and at the same time improving agricultural practices to lessen environmental impact while using scarce natural resources efficiently are the main challenges facing the global agricultural sector in the coming decades. The adoption of microbiome-based practices all along the agrifood system represents a key to address this challenge (Schlaeppi and Bulgarelli, 2015; Suman et al., 2022). The term “microbiome” refers to the whole of microbiota (i.e., the communities of microorganisms colonizing a specific environment) together with the “theater of activity” comprising: (i) peptides, lipids, polysaccharides, DNA and RNA possessed by the microorganisms (their structural elements); (ii) metabolites produced by microorganisms; and (iii) the conditions of the environment in which the microorganisms live (Berg et al., 2020).

Soil, representing the basis of food production, is an essential component for supporting food security through the wide range of ecosystem services it provides. Soil microorganisms contribute significantly to biodiversity and productivity in agroecosystems by participating in nutrient cycles and the decomposition of organic matter (Daunoras et al., 2024; Iqbal et al., 2023), and regulating, supporting, and provisioning services (Anikwe and Ife, 2023; Saccá et al., 2017). Soil and plant microbiomes (rhizospheric, endophytic, and epiphytic) play an important role in plant growth and development, and also in soil health as they provide the plant with a secondary genome that supplies key ecological functions and benefits to the host. Microbiomes play an important role in the management of phyopathogens by enhancing stress tolerance and thus providing an adaptive advantage; they mediate several plant functional traits; they influence plant phenotypic plasticity, and are critical in ensuring the quality and safety of plant primary production, including fruits and related processed foods (Compant et al., 2019; Panke-Buisse et al., 2015; Timmusk et al., 2017). Utilizing the functional potential of soil and plant microbiomes may lead to reduced chemical inputs, increased quality and safety of crops and food products, while increasing the provision of beneficial ecosystem functions for the environment and human health (Chaparro et al., 2012; Gopal and Gupta, 2016; Song et al., 2020).

**Figure 1:**
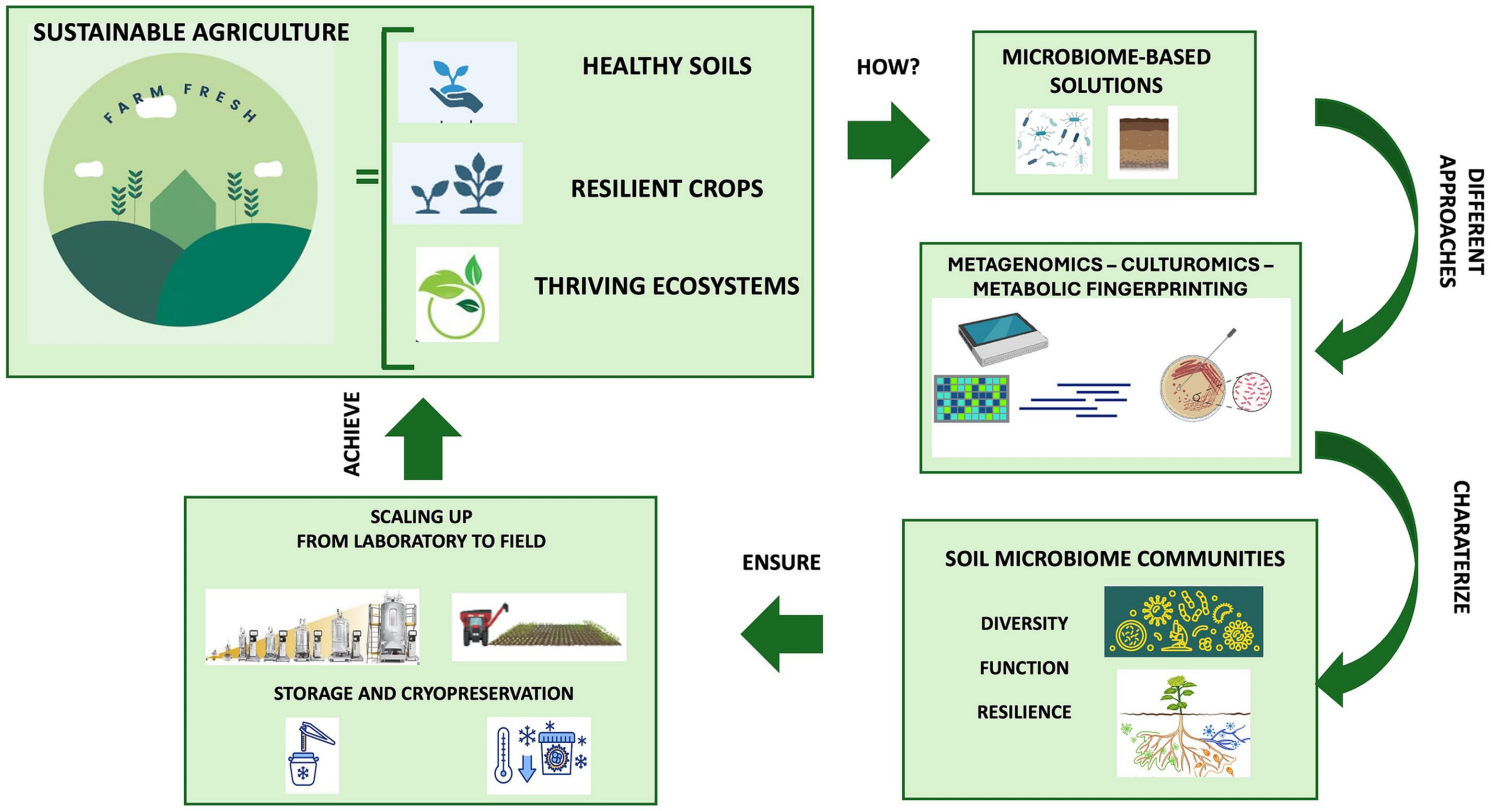
The role of soil microbiome for sustainable agriculture: from selection to preservation and application.

Utilizing microorganisms as biofertilizers is a promising natural-based strategy as they possess multiple nutrient-sequestering and plant-growth-promoting characteristics and can transfer high levels of minerals into plant roots. In agriculture, the use of beneficial microbial consortia, capable of promoting plant growth resistance to biotic (i.e., pest and disease) and abiotic (i.e., drought, flooding, salinity, and nutrient) stress, might help address the challenges posed by modern agriculture (Woo and Pepe, 2018). Today, synthetic and natural communities of different microorganisms with a synergistic activity are constantly being developed and tested (Andreeva and Kozhevin, 2014; de Souza et al., 2020; Tabacchioni et al., 2021; Yin et al., 2022; Hett et al., 2023; Neuhoff et al., 2024). Compared to a single-species inoculum, a multi-species inoculum has the potential to more efficiently increase the growth and yield of plants and improves the availability of minerals and nutrients thanks to its multifunctionality and stability (Liu et al., 2023). Microbiome-assisted sustainable agriculture, therefore, represents a valid strategy for ensuring the health and productivity of plants, thereby influencing the entire food chain and, consequently, the microbiome and human health within the “One Health” framework (Banerjee and van der Heijden, 2023). A better comprehension of soil microbiome composition and function is crucial for the development of tailored microbiome-based formulations under changing climate conditions. The new approaches available (metagenomics, culturomics and metabolic fingerprinting) allow for characterizing the diversity, function and resilience of soil microbial communities, providing insights into their functional potential and fundamental ecological roles for environmental balance. This review delves into the knowledge and techniques applied for understanding the soil microbial diversity and function, highlighting the role of omics strategies for soil health evaluation and how microbiome-based technologies enable us to guarantee sustainable agriculture ranging from the selection to the scaling-up production, preservation and application (Figure 1). Ensuring the scaling up of beneficial microbial consortia from the laboratory to the field allows us to give robustness and replicability to the process, just as the storage and cryopreservation processes of microbial communities are fundamental for long-term preservation and use.

## 2 The plant growth promoting microorganisms and their role for sustainable agriculture

Soils are characterized by the existence of so-called microbial hotspots (i.e., the rhizosphere, drilosphere, or detritusphere) colonized by a fraction of actively microorganisms that is 2–20 times higher than in bulk soil, making temporal changes in microbiome structure and function much more dynamic than sites with less microbial activity (Kuzyakov and Blagodatskaya, 2015). The rhizosphere is a hot spot for microbial growth and a source of beneficial microorganisms for sustainable agriculture (Lynch, 1990; Chamkhi et al., 2022). In the rhizosphere, various microorganisms including bacteria, fungi, viruses, actinomycetes, cyanobacteria, and protozoa thrive due to the diverse nutrients and organic components present. They perform numerous functions, such as biodegradation, conservation of soil structure, and cycling of biogenic elements, that provide essential nutrients to plants. The interactions between microorganisms and plant roots are fundamental for ecosystem functioning. Through these interactions, plants can integrate into the soil, absorb water and ions, and store nutrients (Wu L. et al., 2023). Microbial interactions with roots can vary in nature and have profound implications for plant nutrition and growth, enhancing plant resistance and tolerance to both biotic and abiotic stresses.

Plant growth-promoting rhizobacteria (PGPR) play an important role in promoting soil fertility and improving plant health through their ability to improve crop productivity and nutritional quality. These microorganisms can exert their action through both direct and indirect mechanisms. Direct mechanisms refer, for example, to the mobilization by microorganisms of poorly available nutrient sources (such as recalcitrant soil phosphates), to nitrogen fixation, to the production by bacteria of molecules mimicking phytohormones (e.g., auxins, cytokinins and gibberellins) or enzymes (e.g., 1-aminocyclopropan-1-carboxylate deaminase) that modulate plant hormonal production by degrading the stress-related hormones, and to promote plant growth by improving nutrient absorption and stress tolerance (Compant et al., 2019). Indirect mechanisms may involve controlling plant pathogens by stimulating plant defense mechanisms or suppressing them antagonistically through the production of antibiotics, lytic enzymes, and siderophores. Other bacteria protect plants by modulating levels of plant hormones and inducing systemic resistance (Berg and Koskella, 2018). Furthermore, microorganisms are able to program the plant’s immune system by activating its defense mechanisms against pathogens with an action comparable to that of vaccines, helping the plant to acquire resistance and defend itself from pathogens (Yu et al., 2022). However, under open field conditions, numerous biotic and abiotic stresses can hinder the PGPR effectiveness and reproducibility in promoting plant growth, thereby limiting their application in agriculture.

Plant growth promoting microbes (PGPMs) action can change considerably depending on the microorganism used, plant species, soil type, inoculum density and environmental conditions. Studies of natural populations suggest that groups of microbes with distinct functional niches play critical roles in the adhesion and adsorption of organic nutrients, as well as in the breakdown of organic residues and incorporation into the soil. Several examples of positive plant-microbe interactions include plant-growth promoting rhizobacteria (PGPR) belonging to the genera *Pseudomonas*, *Burkholderia*, *Bacillus*, *Azotobacter*, *Serratia* and *Azospirillum* capable of improving the availability of nutrients in the soil, the absorption and assimilation of nutrients by plants, as well as to support the nitrogen cycle and protect plants from diseases (de Andrade et al., 2023). For example, *Azotobacter* spp. can directly influence nutrition in agroecosystems through the fixation of nitrogen (a vital element for plants), thereby increasing its level in the soil (Aasfar et al., 2021). The ability of these bacteria to form cysts in the soil allows long-term nitrogen conservation and tolerance to drought and high salt stress (Vacheron et al., 2013; Viscardi et al., 2016). *Azotobacter* strains are found in numerous active ingredients marketed as biofertilizers. Furthermore, the ability of *Azotobacter* spp. to secrete substances that promote and regulate plant growth such as phytohormones, vitamins and antifungal metabolites has been studied. Phosphate solubilization (Wani et al., 2013) and Fe-mobilization (Rizvi and Khan, 2018) have been demonstrated *in vitro* and in soil, even under abiotic stress conditions (Van Oosten et al., 2018).

In addition, rhizosphere fungal communities play a key role in agriculture ecosystems due to the positive fungus-plant interactions such as those established by arbuscular mycorrhizal fungi (AMF) which constitute a group of root obligate biotrophs capable of establishing a mutualistic symbiosis with most vascular plant species (Rouphael et al., 2015; George and Ray, 2023). AMF are mainly involved in phosphorus mobilization, enhance nutrient provision in exchange for carbon and increase the plant’s ability to absorb water and nutrients, exerting an important role in regulating growth, and enhancing productivity especially under abiotic stresses (Wahab et al., 2023). Besides, the free-living opportunistic fungi like *Trichoderma* spp. are common in soil and plant root systems. They exert multiple beneficial effects on plants and represent the best candidates for use in agriculture as biofertilizers for the biological control of plant pathogens, mainly fungal phytopathogens, as demonstrated by their presence in several agricultural commercial biofertilizers (Woo et al., 2014; Poveda and Eugui, 2022).

Understanding the complex interactions between plant roots and microbial communities in the rhizosphere has fueled research into the role and application of soil microorganisms in agriculture as biofertilizer and/or biocontrol agent (Solomon et al., 2024). PGPRs, through their complex direct and indirect mechanisms, offer promising alternatives to traditional agrochemical products in organic farming and facilitate the transition from conventional to sustainable agriculture. Despite their potential, the high complexity of ecosystems and the influence of both biotic and abiotic factors can negatively impact their efficacy. Nevertheless, recent advancements in omics sciences are expected to enhance our understanding of plant-soil-microorganism interactions, leading to improved knowledge of PGPR use. This progress is essential for improving crop stability, productivity, and overall agricultural sustainability.

## 3 Molecular and bioinformatic approaches to monitor microbial diversity

**Figure 2:**
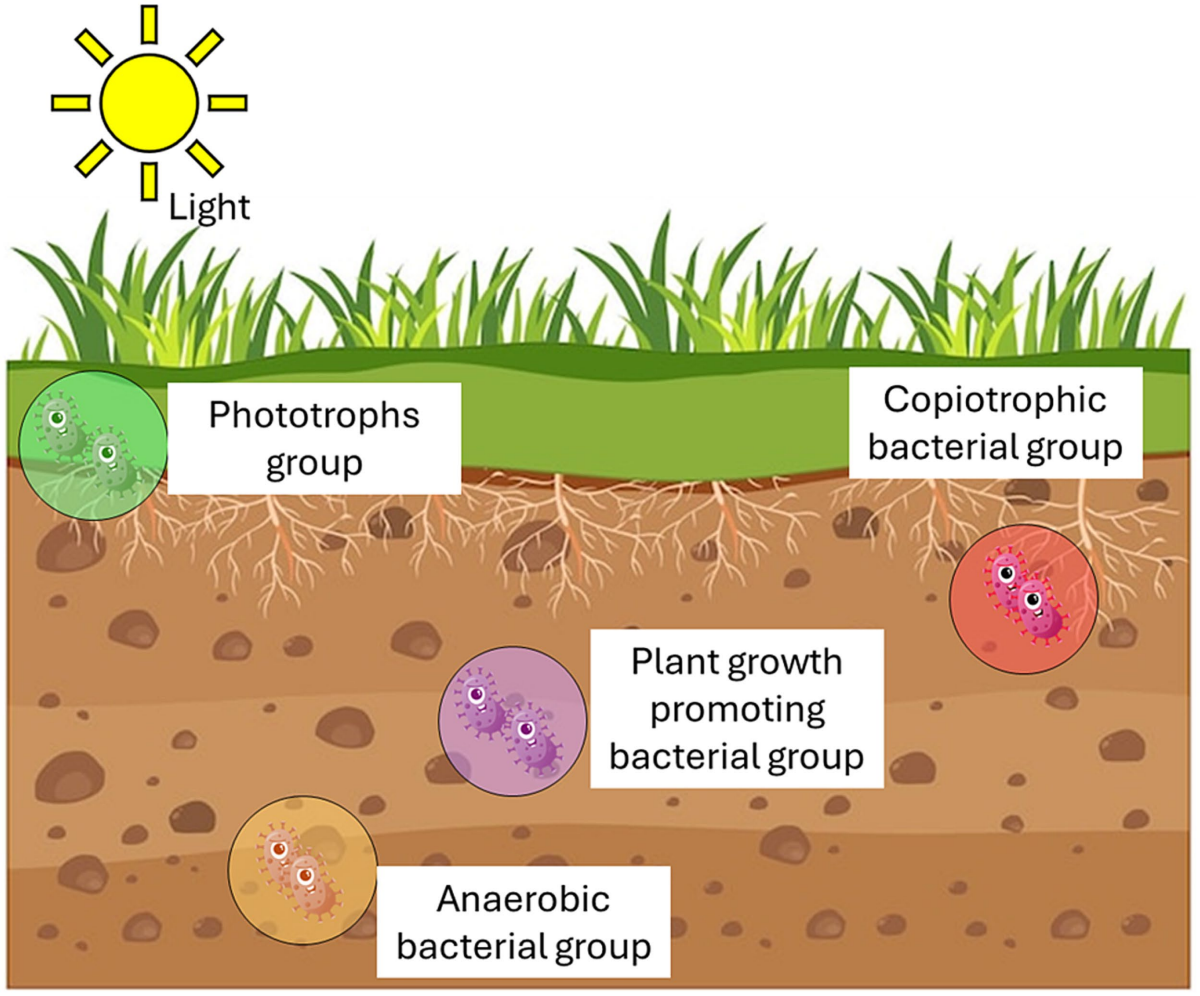
Microbial functional guilds/groups in soil.

Soil constitutes a complex ecosystem, housing various microenvironments characterized by diverse physicochemical gradients and intermittent environmental factors (Voroney et al., 2024). Microbes adapt to these niches, forming consortia with distinct boundaries, interacting among themselves and with other soil organisms through a complex web of relationships that include competition, cooperation, nutrient exchange, and signaling, ultimately contributing to the stability, productivity, and resilience of the soil ecosystems (Wu D. et al., 2023). Studies underscore the influence of soil structure and spatial isolation on microbial diversity and community composition. Examination of bacterial distribution in different soil fertilization regimes revealed that over 80% inhabit micropores within stable soil micro-aggregates (2–20 μm) (Gupta and Germida, 2015; Li et al., 2019). These micropores provide optimal conditions for microbial growth, including water and substrate availability, gas diffusion, and protection against predators (Erktan et al., 2020). Particle size exerts a greater impact on microbial diversity and community structure compared to factors like bulk pH and organic compound input (Philippot et al., 2024). Research demonstrates higher microbial diversity in fractions containing small soil particles, indicating a particle-specific microbial community composition (Heckman et al., 2022). Microbial diversity in soil ecosystems surpasses that of eukaryotic organisms by a significant margin. A single gram of soil can host up to 10 billion microorganisms spanning thousands of species (Pepper and Brooks, 2021). However, less than 1% of these microorganisms observed under the microscope are cultivated and characterized, rendering soil ecosystems largely unexplored. Microbial diversity encompasses complexity and variability across various biological levels, including genetic variability within species (taxa), as well as the richness and evenness of taxa and functional groups (guilds) within communities (Figure 2) (Torsvik and Øvreås, 2002; Roberts, 2019).

The primary challenge in microbiology today lies in connecting phylogeny with function (Berg et al., 2020; Nagy et al., 2020). While methods based on *16S rRNA* gene analysis offer extensive information about the taxa present in an environment, they offer limited insights into the functional roles of each phylogenetic group (Jones et al., 2021; Djemiel et al., 2022b). Metagenomic analysis provides some functional information through genomic sequences and trait expression, but additional methods are necessary to link specific functions with the responsible groups. Quantitative and comparative analyses of expressed *16S rRNA* genes and genes encoding key enzymes, in conjunction with environmental factors, can provide insights into the phylogeny and ecology of functional bacterial groups involved in processes such as denitrification, nitrification, and methane oxidation (Blazewicz et al., 2013). Integrated comparative analyses of core housekeeping genes such as *16S rRNA* and functional genes offer insights into both the phylogenetic diversity and potential functional diversity of microbial communities (Clark et al., 2021; Youngblut et al., 2022). Additionally, it is crucial to comprehend how microbial cells are regulated under diverse conditions like carbon supply, energy source availability, and electron acceptor availability (Yin et al., 2021). This understanding helps in deciphering microbial community responses to environmental fluctuations.

The inability to culture many microorganisms hampers our ability to understand the physiological and ecological foundations underlying the observed spatio-temporal patterns in microbial community structure. Meta-omics offers a solution by recovering genomic, transcribed, and expressed gene information directly from the environment, bypassing the need for cultivation (Tripathi and Nailwal, 2020; Viacava et al., 2022). Multi-omics studies of extreme environment communities have highlighted the prevalence of gene families implicated in energy conservation, carbon fixation, nitrogen metabolism, and resistance to extreme environmental stresses (Li and Wen, 2021). Moreover, comparative omics analyses have been employed to explore functional dynamics across various spatio-temporal scales, targeting microbial communities from geographically distinct systems, specific environmental gradients, diverse ecological niches or lifestyles (e.g., free-living and biofilm growth), as well as time series data (Gamalero et al., 2022; Cabrol et al., 2023).

Indeed, the analysis of microbial community structure and function has now become commonplace thanks to targeted and untargeted sequencing methods, as well as the advent of third-generation sequencing technologies (Kumar et al., 2021; Satam et al., 2023). In fact, the structure of the microbial community typically obtained through targeted methods, i.e., sequencing the *16S rRNA*, allows for the estimation of diversity indices (Straub et al., 2020; Regueira-Iglesias et al., 2023). A diversity index is a quantitative metric that indicates the variety of different types, such as species, within a dataset or community (Hill et al., 2003; Cameron et al., 2021). These indices provide statistical representations of biodiversity, encompassing aspects like richness, evenness, and dominance (Chakraborty, 2021; Díaz and Malhi, 2022). While diversity indices are commonly used in ecology, the types of interest can extend beyond species to include categories like genera, families, functional types, or haplotypes.

Biologists have devised three quantitative metrics to assess and compare species diversity (Walters and Martiny, 2020). Alpha diversity, often referred to as species richness, denotes the total count of species within a specific biological community, like a lake or a forest. Gamma diversity encompasses the overall number of species present across an extensive region, such as a mountain range or continent, encompassing multiple ecosystems. Beta diversity serves as a bridge between alpha and gamma diversity. It quantifies the rate at which species composition varies across a given region and is computed by dividing gamma diversity by alpha diversity.

While diversity indices are commonly used to assess diversity in microbial communities, they were originally developed for “macro”-organisms within ecology. The microbial richness often observed in soil microbial communities can be challenging to manage using traditional calculations. As a result, various bioinformatic tools have been created to enhance the estimation of microbial diversity using data derived from Next Generation Sequencing (NGS) techniques (Cameron et al., 2021). For example, the R packages *Phyloseq*^1^ and *vegan*^2^ are designed to manipulate those data. *Phyloseq* is designed for analyzing microbiome data, allowing for the import, manipulation, and visualization of microbial community data. It provides functions for calculating diversity indices, performing ordination analysis, and generating publication-quality graphics. *Vegan* is used for ecological analysis, including diversity analysis of microbial communities. It provides functions for calculating various diversity indices, performing ordination analysis, and visualizing microbial community data.

There are two primary methods for studying microbial communities with NGS: *targeted* (or amplicon) gene studies and *untargeted* whole-genome shotgun (WGS) metagenomics. Targeted analyses focus on sequencing specific gene regions to reveal the diversity and composition of particular taxonomic groups in an environmental sample. As already mentioned, key marker genes in microbial ecology include the *16S rRNA* gene for archaea and bacteria, the *internal transcribed spacer* (*ITS*) region for fungi, and the *18S rRNA* gene for eukaryotes. On the other hand, WGS metagenomics sequences all genomes within an environmental sample, allowing for the analysis of biodiversity and functional capabilities of the microbial community. This method enables the characterization of the full diversity of a habitat, including archaea, bacteria, eukaryotes, viruses, and plasmids, as well as their gene content. Since their development, WGS metagenomics and targeted gene analyses have set new standards in microbial ecology, extensively used in combination with NGS technologies to characterize microbial communities. The primary advantage of WGS metagenomics over targeted sequencing is its ability to characterize both the genetic and genomic diversity of the analyzed community, as well as the potential and novel functions within the community. With sufficient sequencing depth, complete genomes can be reconstructed from metagenomic data, offering valuable insights into the genomic diversity of microbial ecosystems and enabling the recovery of draft genomes from uncultured organisms. While recent methods can classify marker gene sequences down to taxonomic levels below genus, differentiating between genomes with highly similar marker gene regions remains challenging. WGS metagenomics, however, allows for more precise taxonomic assignments at the species and strain levels. Additionally, WGS is less prone to the PCR biases commonly associated with marker gene amplification, such as the influence of cycle number, primer selection, and the choice of hyper-variable regions. Despite its advantages, WGS metagenomics can still be affected by biases in the metagenomic output, particularly when whole-genome amplification protocols are used for low-concentration DNA samples (Pérez-Cobas et al., 2020). Therefore, monitoring soil microbiome diversity using NGS-based molecular approaches is undoubtedly one of the most widely used and effective strategies currently available. However, as technology continues to evolve, so does the ability to accurately define soil microbiome diversity. In fact, with the advent of NGS, it has become possible to identify many non-cultivable microorganisms, significantly expanding our knowledge of microbial classes. Despite this, the identification of species and strains was not possible except with WGS approaches, which are extremely costly and thus not applicable in routine laboratory settings. At the same time, the advent of third-generation technologies is slowly helping to bridge this gap, as the ability to sequence long-reads increasingly allows for species-level identification even with targeted approaches. Despite everything, the taxonomic identification of microorganisms does not completely define the microbiome, as it is often characterized more by its functions than by its composition. Different microorganisms from various groups (or guilds) can perform the same function. Therefore, it is essential to combine the molecular analysis of the microbiome’s genome with other analyses.

## 4 Culturomics-based approaches and metabolic fingerprinting

Within the genomic era, characterized by a highly descriptive and fast output of analytic data fixed in time and space, culturomics has re-appeared from the past bringing innovations to overcome the main disadvantage of the other omics: the supply of living samples and, therefore, the downstream possibilities of microorganisms’ study across the time and space continuum at a biochemical level to unveil physiological traits that have not yet been identified under various growing conditions (Liu S. et al., 2022). The development of culturomics has long been hindered by the concept that most members of a microbiome (99%) are uncultivable (Martiny, 2019). However, a new concept has been introduced suggesting that most microorganisms are culturable under a strict replication of their ecological niche (Lagier et al., 2016). In the context of the soil microbiome, culturomics is of utmost importance to help close the missing gaps between identification and functionality, since the majority of metagenomic sequencing data remains currently unassigned (Sood et al., 2021). Soil culturomics could further help identify new soil health indicators and, when sampled from extreme environments, it could further serve as a reservoir of essential enzymes with potential applications in biotechnology and industry (Rinke et al., 2013).

Traditional culture-dependent techniques have focused on using nutrient-rich media, favoring the growth and study of fast-growing and copiotrophic species (r-strategists), at the expense of slow-growing and oligotrophic species (k-strategist) (Ji et al., 2024). To enhance the cultivation of previously unculturable bacteria, environmental modifications have been useful, for instance, extending the incubation period for slow-growing species (Janssen et al., 2002; Sait et al., 2002; Joseph et al., 2003; Stevenson et al., 2004; Davis et al., 2005), lowering temperatures (20–25°C) to decrease metabolic rates and the production of inhibitory compounds (Janssen, 2008), optimizing the pH to the environmental one (Sait et al., 2006), and reducing inoculum size (Sangwan et al., 2004; Davis et al., 2005).

In recent years, significant progress has been made in cultivation-based techniques through modifications of culture media to better mimic their natural habitats by using diluted nutrient media (Watve et al., 2020; Janssen et al., 2002) or specialized media formulations. In this context, multiple media have been developed such as the VL55 medium, mimicking the low concentration of inorganic ions found in soil (Joseph et al., 2003; Stevenson et al., 2004) or media supplemented with specific substrates. Considering soil, useful substrate and media additives can range from multiple C-sources, (e.g., xylan) (Davis et al., 2005), polymer mixture, enzymes to counteract the formation of reactive oxygen species (Stevenson et al., 2004), nutritional supplements (e.g., polyamines) (Becerra-Rivera et al., 2018) or signaling molecules (e.g., quorum-sensing signaling compounds which can improve the incidence of colony formation) (Stevenson et al., 2004). Utilizing media enhanced with various plant-based or soil extracts has further shown encouraging outcomes in growing and isolating rhizosphere and soil microorganisms (Mourad et al., 2018; Nguyen et al., 2018). Furthermore, selective culture media (e.g., nitrogen free media to screen for nitrogen fixing bacteria or media containing antibiotics) can facilitate the targeting of specific bacteria and guilds (Baldani et al., 2014; McLain et al., 2016; Zuluaga et al., 2020).

Community culture and co-culture methods are a further tool to enhance the isolation of rare and novel bacterial species. The growth of multiple bacterial cells in proximity fosters interactions if they rely on another microbe for growth (e.g., reciprocal exchange of metabolic substrates) (Xian et al., 2020; Boilattabi et al., 2021). Co-culturing can be also up-scaled by using devices (e.g., microscale microbial incubators, micro-petri dishes, microfluidic platforms, or agarose-based microwell chips) that enable simultaneous growth in individual compartments allowing for the exchange of metabolites and essential substances (Kapinusova et al., 2023). Various *in situ* cultivation techniques have been further developed to obtain unculturable microorganisms directly from their natural environment (Sarhan et al., 2019). Soil substrate membrane provide a simulated environment enabling the isolation of target microorganism onto polycarbonate membranes placed over culture plates containing a soil suspension and under controlled conditions (Svenning et al., 2003; Ferrari et al., 2005; Ferrari et al., 2008). On the other hand, diffusion chambers (an agar-matrix pre-inoculated with microbes and sandwiched between two 0.03 μm pore-size membranes and then placed in soil), and their fungal variant, as well as the microbial trap (Gavrish et al., 2008; Lewis et al., 2010), prevent the entry of airborne contaminants and other bacteria while allowing the passage of biotic and abiotic factors, thus facilitating co-dependent interactions (Lewis et al., 2010; Kakumanu and Williams, 2012). Diffusion chambers have been further up scaled to diffusion bioreactors using large volumes of liquid media to enhance the enrichment of soil bacteria (Chaudhary et al., 2019).

The approach of “lab on a chip,” another evolution of the diffusion chambers, has also been essential for the development of the new generation culturomics. Isolation chips, or i-chips, utilizing cutting-edge microfluidics technology, extract bacteria from sequentially diluted soil samples and deposit them into miniature diffusion chambers (only a few for each chamber) which are then reintegrated into natural soil for further incubation, thus facilitating the isolation of novel soil bacteria and enabling “semi *in-vivo*” interactions studies (Berdy et al., 2017; Gurusinghe et al., 2019). Sorting and selecting cells based on their size, shape, or other distinguishing features before cultivation can further speed up the whole isolation process. Sorting leads to a more efficient cultivation, especially of slow growing microorganisms, by partitioning the overall microbial population into distinct subgroups containing similar microorganisms using specialized equipment (e.g., optical tweezers, flow cytometry combined with sorting cell assays, microdroplet encapsulation) (Zengler et al., 2005; Kumar et al., 2020). The use of a wide array of increasingly more specific media can lead to an optimized culture-dependent approach only when coupled with new high-throughput platforms. For example, Huang et al. (2023) engineered a platform called “Culturomics by Automated Microbiome Imaging and Isolation” for robotic strain isolation and genotyping, leveraging machine learning guidance. The system employs an intelligent imaging algorithm to enhance the taxonomic variety of culturomics, surpassing traditional random-picking techniques. This advancement facilitates swift and scalable generation of cultured biobanks tailored to specific needs which is applicable also to the study of soil microbiota and its responses to climate change adaptation (Jansson et al., 2023; Ramasamy et al., 2023).

**Figure 3:**
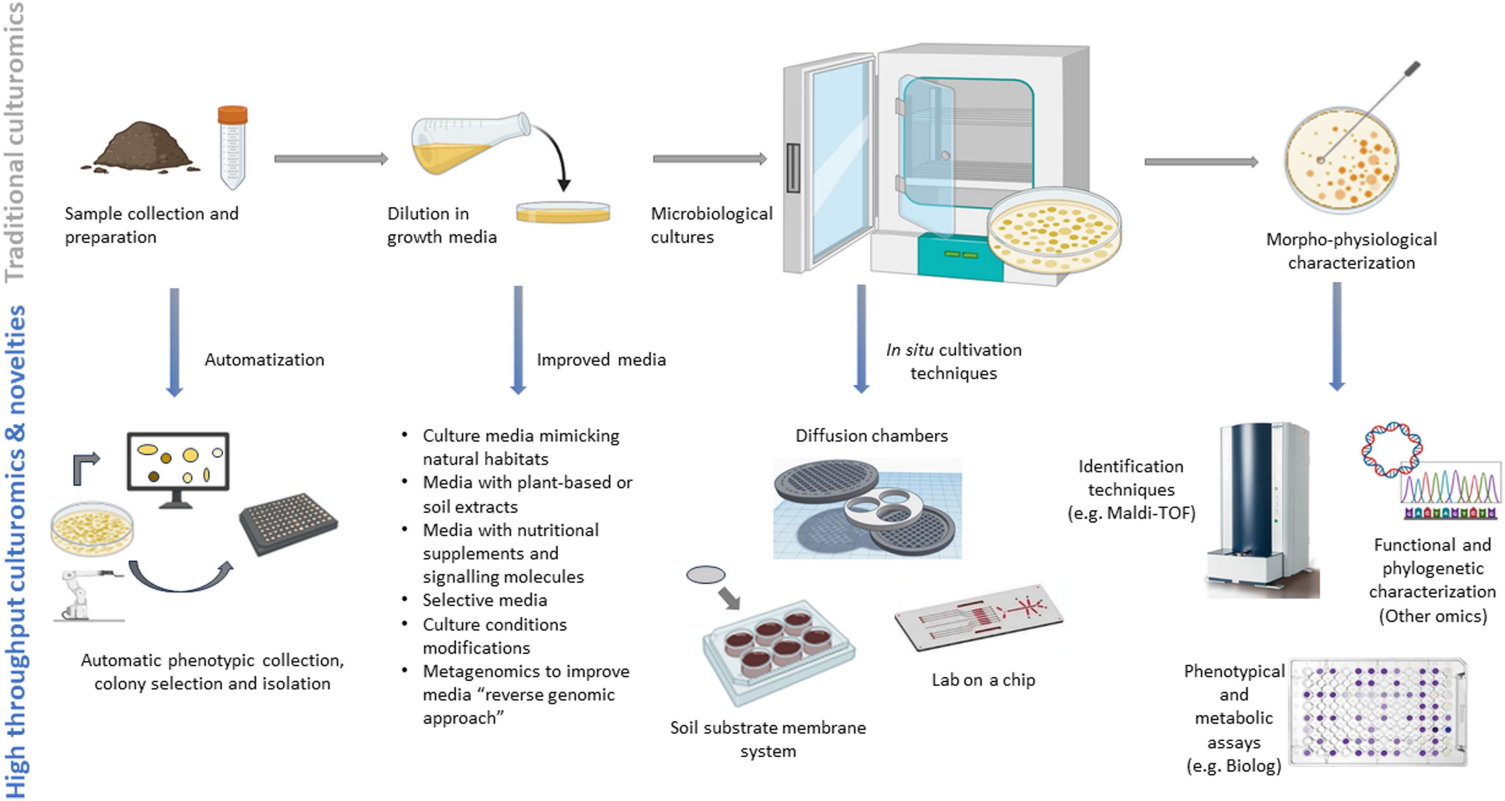
Traditional and novel approaches to culturomics.

The development of new technological advances from the traditional methods have led to the growth of culturomics. Currently, culturomics involves employing high-throughput, large-scale and optimized culture methods combining various selective or enriched culture conditions with identification techniques like matrix-assisted laser desorption ionization-time of flight mass spectrometry (MALDI-TOF MS) and *16S rRNA* or shotgun sequencing to explore the “metagenomics dark matter” capturing maximal diversity (Diakite et al., 2020; Zhang et al., 2021; Li S. et al., 2023) (Figure 3). Conventional untargeted cultivation methods alongside the emerging high-throughput techniques (i.e., culturomics platforms utilizing diverse culture media and screening approaches) have led to the successful cultivation of numerous novel lineages previously inaccessible. Genetic information (genomic data extracted from metagenomes and single-cell genomics) from the targeted uncultured organisms are now employed to tailor the composition of culture media. Through a “reverse genomic approach,” these assembled genomes facilitate accurate predictions of the metabolic pathways (metabolic modeling) which influence culture conditions and, therefore, directly serving as crucial input for *ad hoc* media creation (Sood et al., 2021; Liu S. et al., 2022). Furthermore, media that mimic *in-vivo* nutritional or environmental soil conditions are continuously improved with the bioprospecting for novel compounds, facilitating the development of “unconventional” culturing solutions therefore avoiding the re-discovery of the same microorganisms (Dror et al., 2020).

The coupling of culturomics and metabolic fingerprinting will further have a pivotal role in deciphering microbial diversity and metabolic functions, improving the time-consuming use of phenotypical and metabolic assays on petri dishes. In this context, the Biolog Ecoplates^TM^ system can help screen hundreds of diverse substrates by measuring the consumption of substrates under controlled conditions, allowing for a comprehensively characterization of cellular metabolism, growth patterns, and evaluating the functional diversity of microbial communities (Koner et al., 2022). Other techniques that can characterize the metabolic profiles of the microbes and the production of metabolites involve techniques such as gas chromatography-mass spectrometry (GC-MS), liquid chromatography-mass spectrometry (LC-MS), or nuclear magnetic resonance (NMR) spectroscopy, and especially the MALDI-TOF that can permit to obtain a protein fingerprint or profile unique to each microorganism (Santos et al., 2016). Traditional and high-throughput culturomics methods are shown in Figure 3. Novel methodologies are consistently being suggested for the isolation of bacteria that were previously uncultivated, proving highly effective for targeted isolation purposes. Nevertheless, there remains a significant portion of the tree of life that has yet to be successfully cultivated. The potential for isolating microbes guided by metagenomics is vast, offering a considerable boost by reducing the overall time required to isolate specific targets and initiate their cultivation. Future challenges will focus on utilizing culture-independent genetic data for high-throughput targeted cultivation, coupled with advancements in cultivation techniques, which may lead to groundbreaking discoveries in capturing the uncultured majority (Kapinusova et al., 2023). The integration of multiple omics should place significant focus on unraveling the functional mechanisms behind crucial soil processes and cycles which are vital for the provision of ecosystem services and for introducing innovative metrics to assess soil health (Brown et al., 2024).

## 5 Potential of omics to provide soil health indicators

Microorganisms provide several functions in sustainable crop production and soil health, including decomposition of organic matter, biodegradation of environmental pollutants, carbon sequestration, preservation of soil structure, suppression of pests and pathogens, regulation of soil fertility and circulation of biogenic elements that supply nutrients to plants. For this reason, biological indicators—defined as a single or a set of variables used to represent or infer a specific aspect of soil health—are increasingly gaining importance in the assessment of soil health and quality (Lutz et al., 2023). It is therefore necessary to deepen the study and comprehending effective soil microbial indicators, alongside physical and chemical indicators, to monitor soil conditions. Land-use alterations and agricultural practices significantly influence the soil microbiome by modifying the soil’s physical and chemical characteristics. Additionally, agricultural management techniques such as tillage, pesticide use, and fertilizer application directly impact soil biodiversity by altering these properties (Gupta et al., 2018). In recent years, significant progress has been made in developing novel soil health indicators, and using existing techniques in innovative ways to provide comprehensive insights into soil quality and functionality (Souza et al., 2015). The state of the art in soil health indicators reflects a profound evolution in our understanding of soil ecosystems. Traditionally, soil health assessments relied particularly on physical and chemical, indicators, such as soil texture, soil structure, bulk density, porosity, water holding capacity, infiltration rate, aggregate stability (physical), pH, electrical conductivity, cation exchange capacity (CEC), nutrient content, organic matter content, soil carbon (chemical) (Table 1), and in a minor extent on biological indicators such as microbial biomass, earthworm population, root health and presence of pathogens.

**Table 1:** List of physical and chemical soil indicators, and their lacking points.

Type of indicator	Information	Purpose	Limitations
Physical	Soil texture, soil structure, bulk density, porosity, water holding capacity, water infiltration rate, aggregate stability	Key for understanding soil’s physical condition and its ability to support plant growth	Often measured under specific conditions and may not fully capture the variability in soil behavior
Chemical	soil pH, soil electrical conductivity (EC), cation exchange capacity (CEC), soil nutrient content, soil organic matter (SOM) content, soil carbon	Soil’s health, soil fertility, and soil suitability assessment	They often overlook the complex interactions between soil minerals and organic components

Biological indicators, provide insight into the living component of the soil, and these play key roles in the sustainability of soil by keeping essential functions in soil health, such as: the decomposition of soil organic matter, nutrient cycling, soil pollutant degradation and stability formation of soil structure. To enhance the implementation of soil health indicators, a more integrative and dynamic approach is needed. Combining culturomics and advanced molecular techniques, such as metagenomics and metabolomics, can offer deeper insights into microbial community structure and functional indicators. Culturomics offers a robust approach for analyzing biological soil health indicators by enabling the cultivation and characterization of diverse microbial communities present in the soil (Jagadesh et al., 2024). One of the primary indicators that can be analyzed through culturomics is microbial diversity and abundance. By employing a wide range of growth media and conditions, culturomics can facilitate the isolation and identification of various bacteria, fungi, and archaea. This approach can reveal the presence of previously uncultured and rare microorganisms, providing a comprehensive understanding of soil microbial biodiversity, which is essential for maintaining soil health and ecosystem stability. Another critical indicator assessable through culturomics is microbial functional diversity (Chicca et al., 2022) i.e., by identifying microorganisms involved in key ecological functions such as nitrogen fixation, phosphorus solubilization, and organic matter decomposition. Moreover, culturomics offers the ability to study microbial interactions and community dynamics. By co-cultivating different microorganisms, researchers can investigate symbiotic relationships, competition, and other interactions that influence soil health. For instance, the interaction between mycorrhizal fungi and plant roots can be studied to understand their role in nutrient uptake and plant growth (Liu H. et al., 2022). These interactions are crucial for maintaining soil structure, fertility, and overall ecosystem function.

Metagenomics provides a powerful tool for analyzing a wide array of biological soil health indicators by enabling the comprehensive assessment of microbial diversity and function without the need for cultivation (Djemiel et al., 2022a; Mendes et al., 2017). One primary indicator that can be analyzed is microbial diversity, including the identification of different types of microorganisms present in the soil. By sequencing soil DNA, metagenomics can reveal the presence of rare and abundant species, providing insights into the overall biodiversity and its correlation with soil health. High microbial diversity is often associated with resilient soil ecosystems capable of maintaining functionality under stress conditions (Philippot et al., 2024; Wu et al., 2022). Another indicator is the functional potential of the soil microbiome, which includes genes involved in nutrient cycling, such as those responsible for nitrogen fixation, nitrification, denitrification, and phosphorus solubilization. Metagenomic analysis can identify these functional genes and their relative abundance, offering insights into the soil’s capacity to support plant growth through efficient nutrient turnover. For instance, the presence and activity levels of *nifH* genes, which encode nitrogenase enzymes, can be used to assess the potential for biological nitrogen fixation in the soil (Jansson and Hofmockel, 2018). Soil health is also influenced by the presence of genes associated with organic matter decomposition and carbon cycling. Metagenomics can detect genes encoding enzymes such as cellulases, ligninases, and chitinases, which play a crucial role in breaking down complex organic molecules into simpler compounds that can be utilized by plants and other microorganisms. The abundance and diversity of these genes provide an indication of the soil’s ability to decompose organic matter and recycle carbon, which is essential for maintaining soil structure and fertility (Carbonetto et al., 2014).

Furthermore, metagenomics allows for the detection of genes associated with pathogen suppression and plant growth promotion. For instance, genes involved in the production of antibiotics, siderophores, and other secondary metabolites can be identified, providing information on the potential for biological control of soil-borne diseases. Additionally, genes related to plant hormone production, such as those involved in the synthesis of indole-3-acetic acid (IAA), can indicate the presence of beneficial microbes that promote plant growth and health (Shi et al., 2017). Lastly, soil metagenomics can reveal the presence and abundance of genes related to the degradation of pollutants and the resilience of the soil microbiome to anthropogenic disturbances. This includes genes involved in the breakdown of pesticides, heavy metals, and other contaminants, which are crucial for assessing the soil’s bioremediation capacity and overall environmental health (Kato et al., 2015). By analyzing these biological indicators, metagenomics provides a comprehensive and detailed picture of soil health, offering valuable insights for sustainable soil management practices.

Metabolomics offers a comprehensive approach to analyzing various biological soil health indicators by profiling the small-molecule metabolites present in the soil environment. One primary indicator is the composition and concentration of soil metabolites, which reflect the metabolic activities of the soil microbiome and plants. By analyzing these metabolites, it is possible to gain insights into the biochemical processes occurring in the soil, such as nutrient cycling, organic matter decomposition, and the synthesis of bioactive compounds. For example, the presence of specific amino acids, organic acids, and fatty acids can indicate microbial activity and the state of soil organic matter decomposition (Yang et al., 2023).

Another crucial indicator assessable through metabolomics is the soil’s nutrient status and availability. Metabolomics can identify and quantify metabolites involved in key nutrient cycles, such as nitrogen, phosphorus, and sulfur. For instance, the detection of metabolites like nitrate, ammonium, and urea provides information on nitrogen cycling processes, while the presence of phosphonates and phosphates indicates phosphorus availability and cycling (Wu et al., 2024). Soil health is also influenced by the presence of stress-related metabolites, which can serve as indicators of environmental stressors affecting the soil microbiome and plant roots. Metabolomics can detect compounds such as osmolytes, antioxidants, and stress-related hormones that are produced in response to abiotic stresses like drought, salinity, and temperature fluctuations. The levels of these metabolites provide insights into the resilience of the soil ecosystem and its capacity to withstand and recover from environmental stresses (Krishnamoorthy et al., 2022). Additionally, metabolomics can reveal the presence and activity of PGPR and other beneficial microorganisms by identifying metabolites involved in plant-microbe interactions. For example, the detection of phytohormones such as IAA, gibberellins, and cytokinins can indicate the presence of PGPR that enhance plant growth and development (Napieraj et al., 2023). Similarly, the identification of siderophores and antibiotics can signal the potential for biological control of soil-borne pathogens and the enhancement of plant health through microbial activity.

Furthermore, soil metabolomics can be used to assess the degradation of organic pollutants and the bioremediation potential of the soil microbiome. By profiling metabolites related to the breakdown of pesticides, hydrocarbons, and other contaminants, researchers can evaluate the effectiveness of bioremediation processes and the capacity of the soil to detoxify harmful substances. This information is critical for understanding the impact of anthropogenic activities on soil health and for developing strategies to mitigate pollution.

**Figure 4:**
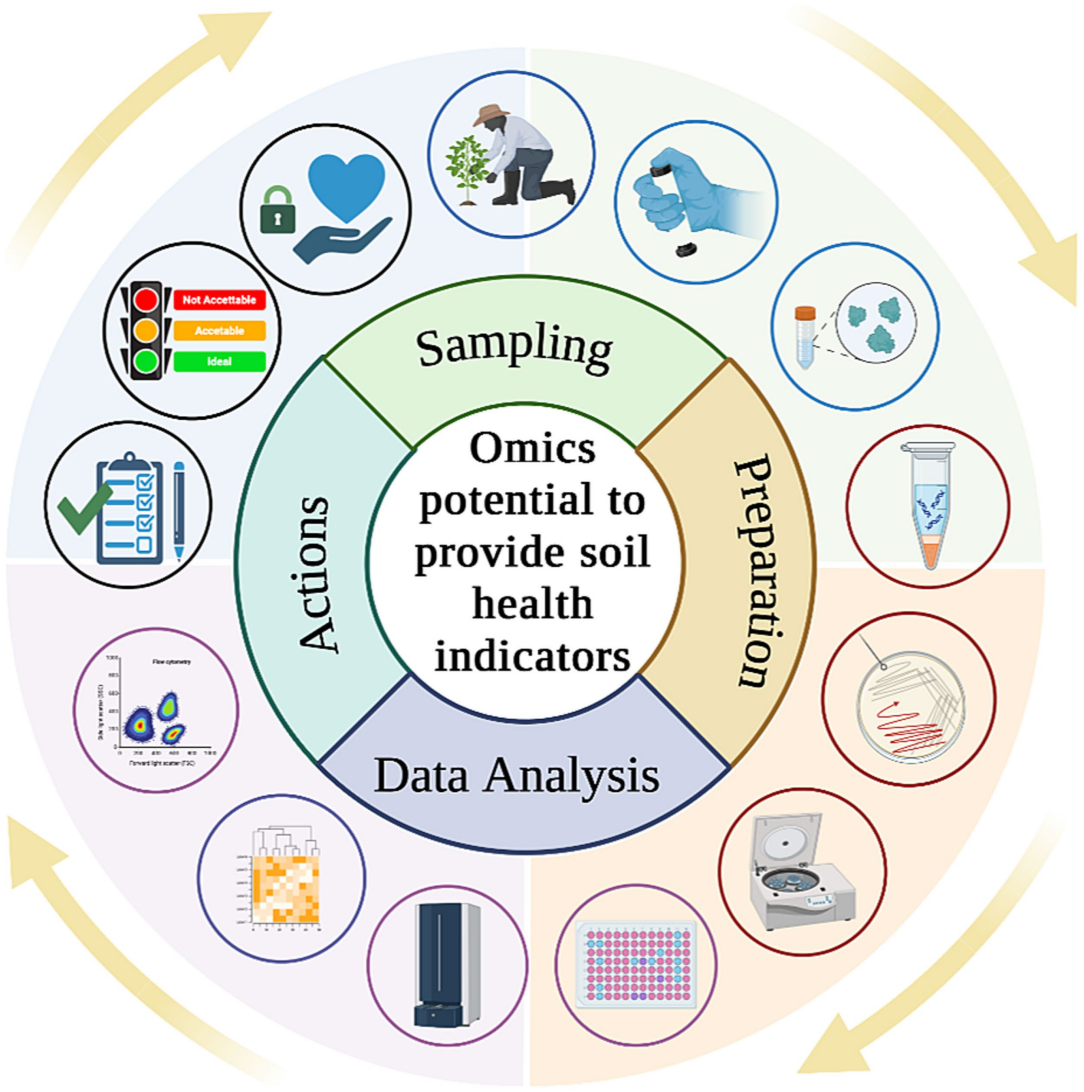
From investigation of soil health indicators to practical actions including policy enhancement and evaluation of new strategies from policymakers.

By examining shifts in microbial community composition in response to different management practices or environmental conditions, it is possible to gain valuable information about soil resilience, disease suppression, and ecosystem stability (Nkongolo and Narendrula-Kotha, 2020). These indicators not only provide insights into soil quality but also offer early warnings of changes in soil health (Fierer et al., 2021), making them valuable tools for detecting subtle shifts in soil conditions that precede more pronounced degradation or disfunction. For this reason, integrating these techniques to assess soil health into existing soil monitoring laws, policymakers could enhance their capacity to protect and preserve this vital resource (Figure 4). Compliance with soil monitoring policies helps governments and stakeholders make informed decisions regarding land management, conservation efforts, and regulatory interventions to maintain soil fertility, prevent degradation, and support sustainable development goals. Moreover, considering advancements in soil science, such as metagenomics, metabolomics and culturomics, it is imperative that legislative frameworks also encompass innovative techniques to ensure thorough soil health assessments.

## 6 Microbiome-based solutions for sustainable agriculture: how to develop beneficial microbial consortia

In recent years, the extraordinary value of the microbiome for plants has been established, and efforts are now being made to understand how it can be harnessed to improve crop production (Kumawat et al., 2022). The use of microbiological systems as environmentally friendly solutions for sustainable agriculture has been widely explored. Microbiome-based solutions represent an innovative and green revolution technology that can ensure greater food production, increase food quality and improve the efficiency of food production systems (Callens et al., 2022). Beneficial microorganisms can be used as biofertilizers to increase crop yield, improve, and restore soil fertility, or as biopesticides to reduce the damage caused by pathogens and pests in agricultural fields, offering an alternative or substitute to decrease the dependency of agriculture on hazardous agrochemicals. The challenge is to identify, isolate and apply beneficial microorganisms able to survive in soil, compete with indigenous microflora, and interact with plants (Romano et al., 2020; Mitter et al., 2021). The effectiveness of microbial inoculants under field conditions is one of the major encountered problems due to the variable environmental factors that often obstacles their successful establishment (O’Callaghan et al., 2022). To reduce the gaps between the efficacy of PGPMs application under controlled environmental conditions and the limited reproducibility of their use under field conditions, guidelines for PGPMs field trial design and implementation have been developed, as well as recommendations for the type and scope of data collection and evaluation (Neuhoff et al., 2024). At the same time strategies have shifted from single-strain inoculation to multi-species consortia for developing inoculants. Two types of approaches were taken into-account to obtain microbial consortia for sustainable agriculture: (i) identification and synthetic assemblage of multi-species strains with different beneficial functions, (SynComs) or (ii) obtainment of complex microbial communities from environmental sample (NatComs).

The SynComs are mixed inoculations of at least three different bacterial strains/members, excluding use of indigenous, natural, or wild microbial communities (Marín et al., 2021). Recently, the use of SynComs has gained great interest for its potential advantages over single species/strains applications in sustainable agriculture (de Souza et al., 2020). The coexistence of non-competitive and diverse microbial species within a consortium can lead to the colonization of a broader range of ecological niches in the plant rhizosphere (Timofeeva et al., 2023). Moreover, the presence of microorganisms with similar or complementary plant beneficial traits can help to improve the efficiency of the inoculant as different plant beneficial functions can be present simultaneously and exhibited by different consortium members (Liu et al., 2023). Recently, the integration of high-throughput sequencing technology coupled with microbial strains and computational genomic analyses of their functional capabilitie has provided the opportunity to identify the core microbes associated with plants and facilitate the tailoring effective SynComs with robust, stable, and predictable behaviours (Höllerer et al., 2024; Jing et al., 2024). As function-based SynCom design strategies, various genomic traits can be considered (i.e., nutrient acquisition, protein secretion systems, biosynthetic potential, secretion of plant-immunostimulating primary metabolites, secretion of phytohormones, antibiotic resistance genes) and different computational frameworks have been developed providing the possibility to design a complex “high-function” community *in silico* (Jing et al., 2024). Two main methods are currently used to define functional microbial synthetic communities: top-down Synthetic Microbial Consortia (SMCs) and bottom-up SMCs. The top-down approach can permit to identify the functional communities by applying the core microbiome concept; therefore, by applying the bottom-up approach, microorganisms with specific plant growth promoting traits can be assembled to design the best microbial combination (Shayanthan et al., 2022).

### 6.1 SynComs

The development of synthetic microbial communities (SynComs) relies on the assembly of microorganisms with similar or different plant growth promoting (PGP) traits, isolated from different sources, acting in a synergist and/or complementary way. The use of SynComs has shown promising results in increasing plant growth and yield, in improving the availability of minerals and nutrients, providing the plants with more balanced nutrition, and in controlling plant diseases (Bradáčová et al., 2020; Liu et al., 2023; Nunes et al., 2024). Prerequisite of this approach is the availability of a large collection of microorganisms well characterized at molecular and phenotypical level. The main challenge in assembling diverse microorganisms is the assessment of their compatibility (Timofeeva et al., 2023). *In vitro* tests are widely used to reveal compatible and incompatible interactions between two microorganisms, but they do not provide a reliable picture of all the possible interactions after the application in field (Nunes et al., 2024). *In vitro* tests with AMF cannot be performed, being these obligate symbionts and not cultivable in synthetic media (Dey and Ghosh, 2022). A binary association assay was used to design a SynCom for the model plant *Arabidopsis thaliana* that led to predictable phenotypes in the host plant (Herrera Paredes et al., 2018). Although using this method is possible to infer causal relationships between selected microorganism and host phenotypes, it requires technological advances to manage high complex communities and increases the chances of missing important community members. Kehe et al. (2019) used the kChip, a microfluidic droplet-based platform, to automatically construct SynComs with all possible microbe combinations using a set of species making this approach more efficient and possible for large scale studies. In the framework of SIMBA project,^3^ three multifunctional SynComs composed of five and six microorganisms belonging to various genera/species and with different PGP traits have been designed, after the assessment of their *in vitro* compatibility, to improve the growth of different crops (Tabacchioni et al., 2021). Greenhouse experiments revealed the potential of these SynComs alone or in combination with biochar and AMF to improve the growth of maize and wheat (Graziano et al., 2022; Hett et al., 2022). Field experiments carried out in Germany, under organic farming, showed the beneficial effect of one of the designed SynComs on maize growth and yield, although this effect was not confirmed in the second year of field experiments (Hett et al., 2023). Moreover, one of the developed SynComs was found to increase tomato marketable production when applied in combination with biochar and AMF in field experiments (Vassura et al., 2023).

Although one limit of the SynComs is the design of microbial consortia with a limited number of members that do not reflect the microbial interactions occurring in the natural environment (Timofeeva et al., 2023), several studies demonstrate the potential of SynComs composed of few microorganisms to improve plant performance. The efficacy of a SynCom containing *Trichoderma atroviride*, *Pseudomonas putida*, and *Bacillus subtilis* to control the incidence of *Rhizoctonia solani* and *Streptomyces* spp. disease of potato in field experiments was demonstrated (Papp et al., 2021). Li J. et al. (2023) developed a microbial consortium, composed of two different bacterial species, able to positively influence the growth of wheat and the soil nitrogen enzyme activity under drought conditions in greenhouse conditions. A microbial consortium composed of three different species was found to reduce the incidence of clubroot disease in broccoli (Moreno-Velandia et al., 2024).

Overall, it was demonstrated that SynComs perform better of single strain inoculants, despite a reduction in efficacy in field settings compared to greenhouse results was observed (Liu et al., 2023). To promote the use of SynComs in agriculture further research on their stability and persistence in the soil, their modes of conservation and applications, and the study of microbe-microbe and plant interactions is needed.

### 6.2 NatComs

Synthetic microbial communities fail to accurately mimic the natural composition since the species and/or strains used as members of the consortium are typically not found together in the same environment, and the environmental system itself due to the use of single carbon sources or mixed liquid cultures for the inoculum. Furthermore, diversity is usually too low to be representative of the entire ecosystem (Čaušević et al., 2022). To overcome the limited SynComs efficacy under field conditions, a recent microbiome-based technology has been explored such as the transplantation of an entire microbiome, i.e., the rhizospheric microbiome (rhizobiome), which should contain functional and active microbiota to protect plant by disease and promote plant growth development. As in clinical settings the fecal microbiome transplant can restore the balance of gut microbial communities and their function (Kaakoush, 2020), in agriculture the rhizobiome microbiome transplant (RMT) represents a new plant microbiome engineering strategy (Orozco-Mosqueda et al., 2023). This technology has received little attention for the difficulty in applying it at large scale, but recent studies have been demonstrated its feasibility, especially for controlling plant disease, by transplanting “protective” microbiomes from resistant to susceptible plants. Choi et al. (2020) used soil microbiota transplant in tomato plants under defined soil conditions to investigate the disease progress of lethal bacterial wilt (BW) disease in tomato. The authors found that soil microbiota transplant affected plant traits, especially BW resistance in tomato, highlighting the efficacy of soil transplantation in influencing plant quantitative traits. Khatri et al. (2022) carried out the transplant of soil from an organic field previously recognized as “disease-suppressive” to a conventional field soil to evaluate the effectiveness of this technique to reduce the disease caused by *R. solani* and *Fusarium oxysporum* in wheat plants. Results revealed that transplant of “disease suppressive soil” reduced disease severity in plants, improved soil nutrient content, increased activity of hydrolytic enzymes, and increased abundance of genes contributing to disease-suppressiveness.

Starting from a single environmental sample, where many different bacteria have a history of co-existence and may have developed synergistic interactions, discrete single species can be isolated, and a systematic screening of all possible strain’s combinations for a phenotype of interest can be performed. It is possible to recover community emergent properties by concurrent microbe isolation from a single environmental sample where different species co-exist and developed interactions and inter-dependency. Following this approach, a microbial consortium consisting of four different bacterial species (*Stenotrophomonas rhizophila*, *Xanthomonas retroflexus*, *Microbacterium oxydans* and *Paenibacillus amylolyticus*) isolated from the same agricultural soil (de la Cruz-Perera et al., 2013) proved capable to produce more biofilm in comparison to the sum of what obtained by the of four strains when grown singularly (Ren et al., 2015). The addition of the microbial consortium prior to forced drought conditions significantly increased the survival rate and biomass of *Arabidopsis* under water shortage, suggesting that this consortium could improve plant tolerance against drought (Yang et al., 2021). A tailored microbial consortium composed by eight indigenous strains was developed as biofertilizer for tomato crop by assembling including different species for the best and complementary plant growth promoting (PGP) traits and reflecting as much as possible the taxonomic composition of the indigenous microbial community structure (Paganin et al., 2024).

However, using conventional method of culturing in liquid suspensions of both SynComs or indigenous-based microbial consortia, it is not possible to capture the natural soil characteristics such as interspecific interactions, ecological niches, emergent community behaviors. Using the top-down approach (NatCom), more diverse and representative, but less controllable communities can be collected directly from natural soil by detaching and purifying the cells from soil particles (Čaušević et al., 2022). Species-rich natural soil inocula can reproducibly be generated, propagated, and maintained from natural microbial mixtures washed from topsoil. The applicability of this latter strategy to open field remains to be further explored in open field studies.

## 7 Preservation of complex communities

The concept of preserving intact samples and microbiomes with retained viability and functionality for future OMICS, cultivation, and application is highly relevant today. Preserving uncultured microbiota and intact microbiomes in as close a state as possible to that originally present in field is crucial in microbial research and of great importance (Bhattacharjee et al., 2022). The study, and therefore preservation, of microbial diversity and metabolic activity is necessary to elucidate microbial community composition, interactions and functional dynamics (van der Heijden and Wagg, 2013). Ideally, both culture dependent and independent molecular methodologies should utilize fresh soil samples as starting material as immediate soil analysis yields an accurate depiction of the microbiome (Wallenius et al., 2010). However, advancements in culture independent techniques revealed that over 90% of microorganisms of biotechnological relevance is not yet cultured nor stored in biobanks (Prakash et al., 2020). To unlock the potential of uncultured organisms, it is necessary to protect microbial biodiversity within samples from disruption, eventually waiting for cultivation methods to be optimized soon. However, analyses on freshly sampled soils are often challenging, especially when dealing with experiments conducted in remote areas, requiring a high number of samples, or necessitating chronological comparisons (Pavlovska et al., 2021).

Protecting microbial biodiversity within a sample from disruption and maintaining the composition and functional potential of its microbiome is therefore a primary concern Several crucial factors come into play to ensure the collection and maintenance of representative samples: sampling protocols, transportation logistics, and storage methodologies. The scientific community is currently focusing on conserving ecosystems without disturbing their microbiomes and losing information. Due to the vast complexity of soil matrices, a consensus on optimal and standard operating procedures to collect and preserve a microbiological sample is still lacking leading to an impossibility of replicability and data comparison. To solve this, problematic several projects are emerging that aim to establish and validate quality standards for microbiomes, testing different preservation approaches to identify the best preservation method that permits to maintain sample integrity (e.g., the Italian SUS-MIRRI.IT project,^4^^,^^5^ the EU-funded MICROBE project with the cooperation of research infrastructure^6^^,^^7^ and the EU microbiome support CSA project^8^) (Ryan et al., 2021).

Several advanced methods for microbiome preservation are available that enable the maintenance of a sample’s properties (Prakash et al., 2020). A novel technique is the Cell Alive System (CAS), originally developed to enhance preservation in the food industry, it utilizes electromagnetic waves to induce oscillation in the water molecules inside cells therefore keeping water molecules in a supercooled state below zero degrees without freezing. Once it reached the desired temperature the sample is then rapidly frozen, preventing the formation of large crystals and maintaining the integrity of the cells (Morono et al., 2015).

One possible strategy could be the application of the long-term preservation methods usually applied for the storage of axenic samples in culture collections, such as cryopreservation and lyophilization, but some limitations may arise. The use of ultra-low temperatures can quickly halt metabolic processes, maintaining physiological conditions in a suspended state (Bajerski et al., 2021; Murray and Gibson, 2022). However, ice-crystal formation and the gradual increment of solutes concentration during freezing can be lethal for microbial cells without cryoprotective agent (Wolkers and Oldenhof, 2015). Similarly, the lyophilization technique can preserve microorganisms in a sort of inactive state but can induce osmotic stresses in biological membranes. Lyoprotectans can be used during the drying process to prevent mechanical damage to the cells (Broeckx et al., 2016), but it is unclear how different microbes behave in terms of viability and functionality when stabilizers are added to the media. Decreasing in survival rates of specific microbial taxa have been demonstrated during freezing or freeze-drying processes (Miyamoto-Shinohara et al., 2000; Alebouyeh et al., 2024) and the effects could be magnified when scaling-up these techniques on complex microbial populations. Numerous studies have shown heterogeneous results on the impact of different storage temperatures and preservatives on soil microbial communities (Lauber et al., 2010; Rubin et al., 2013; Brandt et al., 2014; Delavaux et al., 2020; Edwards et al., 2024). It may be that outcomes are also influenced by soil types (e.g., generic surface soil, forest soil, mineral soil, meadow soil), suggesting that each ecosystem’s specific microbiome is selectively susceptible to different treatments. This could result in undesired selection of resistant microorganisms when specific storage techniques are applied, causing compositional shifts that affect community representativeness and abundance in a sample. Furthermore, ecological perturbations and climatic variations have been observed to gradually shrink the core microbiome of any ecological system, raising the possibility of extinction and loss of valuable microbiota components over time.

Until now, no single method, process, preservation protocol, or cryoprotectant works optimally for every kind of sample due to microbial heterogeneity in microbiomes, which respond differently to various preservation methods. This heterogeneity makes it challenging to apply the same protocol or preservation conditions to all samples. The challenges of preserving microbiome samples optimally are significant. Researchers must be aware of the potential for unintentionally and fundamentally altering the functionality and integrity of the microbiome, a dynamic system that changes in response to environmental influences and biotic factors. Removing a single critical microbial component due to a non-optimized storage approach could irreversibly affect the system’s integrity.

## 8 Scaling-up and application

The growing interest in organic agriculture, the increased use of PGPMs in developed countries, as well as the good consumer acceptance towards the use of these microorganisms as an ecological alternative to agrochemical products, is revolutionizing the development of biofertilizers (Ibáñez et al., 2023). Indeed, according to MarketsandMarkets,^9^ the global inoculants market size is projected to reach USD 1.7 billion by 2027, recording a CAGR of 8.1% during the forecast period. To respond to this market demand, the production of biofertilizers must necessarily be economically and ecologically more advantageous than the chemical one. Generally, microbial biomass is produced through fermentative processes using synthetic substrates which represent approximately 60–70% of the overall costs (Cardoso et al., 2020; dos Santos et al., 2022). In fact, the use of very expensive conventional substrates, consisting for example of yeast extract, beef extract, peptone and glucose, may be acceptable for growth tests in flasks or in small volumes but at larger volumes, as in fermenters at pre-industrial or industrial scale would lead to a rapid increase in production costs (Lo et al., 2020; Yuan et al., 2021). Therefore, choosing an appropriate culture medium to produce high quantities of microbial biomass is a binding issue to achieve sustainable production costs (Lo et al., 2020; Vasseur-Coronado et al., 2021). In this context, the development of a low-cost process is the main challenge which pushes the academic world to intensify studies to achieves microbials inoculant production at industrial level (Arora et al., 2016; Santoyo et al., 2021; Vasseur-Coronado et al., 2021; de Andrade et al., 2023; Agbodjato and Babalola, 2024). To reduce fermentation costs, complex raw materials derived from agro-industrial and food waste are mainly used. For example, corn syrup, crude glycerol, distillers’ yeast, molasses, whey, soybean meal, corn liquor (CSL), and starch are widely used (Cantabella et al., 2021). Various primary and secondary metabolites can be produced using as suitable nutrients these relatively inexpensive raw materials to ensure the growth of bacteria.

In the literature there are many studies conducted on a laboratory scale with the aim of evaluating the use of carbon and nitrogen sources derived from agro-industrial waste for the growth of different microorganisms (da Silva Cruz et al., 2020; Gamit et al., 2023; Omara and El-maghraby, 2023; Torres et al., 2023; Akashdeep et al., 2024). Conversely, there is little information regarding the application of low-cost media in both small- and large-scale bioreactors to produce microorganisms that promote plant growth. Table 2 summarizes some studies conducted with the aim of developing low-cost growth substrates to be used in fermentation processes generally carried out in solid state (SSF) or in liquid state (LSF). Magarelli et al. (2022) have developed a process to produce PGPMs by submerged fermentation using the cladode juice of *Opuntia ficus-indica*, reducing both the economic and environmental impacts associated with fermentations. In this research, the scaling up of the growth process was carried out in a 21-liter bioreactor. In a recent work, Bolmanis et al. (2023) reach high concentrations of spores of *Bacillus subitlis* MSCL 897 in 100-liter bioreactors using a low-cost substrate consisting of sugar beet molasses and bean flour. Instead, Elsallam et al. (2021) conducted a study aimed at reducing the costs of industrial production of biomass of the fungus *Trichoderma harzianum*. Specifically, the strain SYA.F4 has been grown in flasks on culture media consisting of different agri-food waste such as potato, onion, garlic, pea, and cabbage peels. Subsequently, using pea peels the process was scaled up into a 7-liter fermenter by using exponential fed-batch mode reaching a high yield in fungal biomass. *T. harzianum* CECT 2929 was also successfully grown in SSF on grass clippings and pruning waste by Ghoreishi et al. (2023). The growth of fungal strain in a 0.5 L cylindrical fermenter permitted to reach after 168 h spore concentrations equal to 3.03 × 10^9^ spore g^−1^ dry matter.

**Table 2:** Studies on scaling up the production process of microorganisms that promote plant growth using agro-industrial waste.

Microorganism	Type of fermentation process	Composition low-cost culture media	Scale bioreactor	References
*Bacillus* spp., *Paenibacillus* spp.	LSF	Food waste and green waste	120 L	Oviedo-Ocaña et al. (2022)
*Bacillus subtilis* CW-S	LSF	Molasses and urea	300 L and 3,000 L	Abuhena et al. (2022)
*Bacillus subtilis* MSCL 897	LSF	Molasses and bean flour	100 L	Bolmanis et al. (2023)
*Azotobacter chroococcum* LS132, *Bacillus amyloliquefaciens* LMG 9814, *Burkholderia ambifaria* MCI*7*, *Pseudomonas fluorescens* DR54 and *Rahnella aquatilis* BB23/T4d	LSF	Opuntia pruning waste	21 L	Magarelli et al. (2022)
*Paenibacillus polymyxa* DSM 742	LSF	Brewers’ spent grains	5.5 L	Didak Ljubas et al. (2022)
*Pseudomonas oryzihabitans* PGP01	LSF	Potato waste (peels and pulps)	2 L	Cantabella et al. (2021)
*Bacillus siamensis* SCFB3-1	LSF	Anaerobic digestate obtained from fruit and vegetable wastes and molasses	5 L	Pastor-Bueis et al. (2017)
*Bacillus subtilis* CGMCC13932	LSF	Tofu processing wastewater from soybeans	100 L	Li et al. (2021)
*Trichoderma harzianum* SYA.F4	LSF	Pea peels	7 L	Elsallam et al. (2021)
*Trichoderma harzianum* CECT 2929	SSF	Grass clippings and pruning waste	0.5 L	Ghoreishi et al. (2023)
*Beauveria bassiana* CECT 20374 and *Trichoderma harzianum* CECT 2929	SSF	Rice husk, apple pomace, whisky draff, beer draff, wheat straw, orange and potato peels	0.5 L	Sala et al. (2021)
*Azotobacter vinelandii* MTCC 1241, *Rhodobacter erythropholis* MTCC 4688, *Bacillus megaterium* NCIM 2054 and *Rhizobium meliloti* NCIM 2757	SSF	Textile sludge and sugarcane bagasse	15 L	Kadam et al. (2024)

Moving from a laboratory scale to a larger scale, some growth parameters such as substrate composition, temperature and pH are easily controlled. Conversely, the best conditions identified to control the oxygen content in the medium are not always efficient when large bioreactors are used. Furthermore, to guarantee the robustness and replicability of the scaling up process it is important to consider the issues relating to the phenotypic dissociation characteristic of some spore-forming bacterial species (Ambrico et al., 2019). In this regard, after identifying the best growth conditions, in small bioreactors it is essential to identify the volumetric oxygen mass transfer coefficient (k_L_a), a specific parameter that determines the rate at which oxygen is transferred from the gaseous to the liquid phase. This coefficient depends not only on the speed and type of stirring in the bioreactor but also on the properties of the media and the geometry of the vessel (Vanags and Suleiko, 2022). The presence of oxygen in the growth substrate is a key factor in aerobic bioprocesses. In bioreactors, the oxygen content is controlled by acting on the agitation, modifying the rotation speed of the turbines, and varying the volumes of air and/or oxygen introduced using a sparger. Therefore, to maintain adequate oxygen supply even in large bioreactors, ensuring the optimal growth of cell populations and the maintenance of their normal metabolism, the scale-up of aerobic fermentations must take place maintaining the k_L_a constant. Over the last few years empirical formulas and several experimental methods have been proposed to determine the value of k_L_a in bioreactors under different operating conditions (Karimi et al., 2013; Trujillo-Roldán et al., 2013; Seidel et al., 2021; Mercado et al., 2023). Trujillo-Roldán et al. (2013) by keeping the k_L_a constant, they managed to scale the growth process of *Azospirillum brasilense* from agitated flasks to 1,000-litre bioreactors. Using an experimental method, the authors determined the k_L_a in 0.5 L flasks and in 10- and 1,000-liter bioreactors as a function of different rotation speeds. Subsequently, they plotted the data in graph and by interpolation they identified the speed of rotation to be adopted in the two larger bioreactors to obtain k_L_a values like that determined in flask in the best growth conditions (k_L_a = 31 h^−1^).

Addedly to the selection of a suitable low-cost culture medium for optimal biomass production and scaling-up process, to achieve an effective and stable formulation is an important issue in inoculant technology. In fact, often when the inoculating microorganisms are used in *in-vivo* assays in a controlled laboratory environment, they can best express their potential. Conversely, their behavior is unpredictable when applied in the open field where they find limiting environmental conditions and an indigenous microbiota to compete with (Shah et al., 2021). These situations vary from crop to crop and from field to field and can cause an inconsistency in the beneficial effect of inoculants, making it much more complex for developers and commercial distributors to provide PGPR inoculants that are effectively applicable under different environmental conditions (Saharan and Nehra, 2011). These situations vary from crop to crop and from field to field and can cause an inconsistency in the beneficial effect of inoculants, making it much more complex for developers and commercial distributors to provide PGPR inoculants that are effectively applicable under different environmental conditions (Saharan and Nehra, 2011). Furthermore, most governments regulate quality standards by imposing a minimum number of viable cells ranging from 10^7^ to 10^9^ colony-forming units per gram for an adequate formulation (Bashan et al., 2014; Malusá and Vassilev, 2014).

Identifying an effective and stable formulation represents an essential aspect to improve the adaptability of microbial inoculants to different environments, promote colonization and ensure survival during storage (Mitter et al., 2021; Naamala and Smith, 2021). The formulations can be both liquid and solid and the latter can be both wet and dehydrated. The choice of formulation type depends on the type of microorganism and application. Generally, liquid ones are used for seed treatment, seedling root dipping and soil irrigation (Dey, 2021). These consist of microorganisms, if possible, in their inactive state, suspended in water, oils, or emulsions with additives to improve their physical, chemical and nutritional properties. The main additives used are natural polymers (e.g., carrageenan, arabic gum, starch, etc.), synthetic polymers (such as polyvinylpyrrolidone), humic acid, horticultural oil, glycerol, glucose and lactose (Bernabeu et al., 2018; Ibáñez et al., 2023). In general, support material for biofertilizers should be non-toxic, widely available, inexpensive, and easy to use (Allouzi et al., 2022). For example, polymers, both natural and synthetic, reduce heat transfer and increase water activity by ensuring a protective microenvironment and the amount of water biologically available to microorganisms (Lobo et al., 2019). Humic acids provide shelter and carbon sources to microorganisms, ensuring a minimum of metabolic activity during storage, reducing the loss of vitality (Sadeq et al., 2023). Gopi et al. (2019) reported that, from a screening of various components useful for the formulation of liquid biofertilizer, trehalose at a concentration of 15 mM was the best performing. Even after 18 months, a significant population of *Azospirillum lipoferum*, *Azotobacter chroococcum*, *Bacillus megaterium* and *Bacillus sporothermodurans* was observed in this formulation. Other researchers have used a combination of trehalose and glycerol to successfully extend the preservation of the bacteria by achieving a biofertilizer shelf-life of 12 months (Chompa et al., 2024). The higher viability of microbial cells found in formulations with trehalose is due to the protective effect of this disaccharide on membrane proteins and to the antioxidant effect guaranteed by the eight hydroxyl groups present in the molecule (Kumaresan and Sivakumar, 2019; Prasad et al., 2023).

While, glycerin, in addition to providing a carbon source to stimulate bacterial cell growth, regulates osmotic pressure and nutrient exchanges at the plasma membrane level (Nasarudin et al., 2020). Horticultural oils can be used to extend the shelf-life of the water in oil emulsion formulation since these compounds are able to envelop the bacterial cell and protect them from dehydration and temperature changes (Allouzi et al., 2022). Furthermore, it has been shown that the presence of oils favors the production of exopolysaccharides as a response to various environmental stresses. Oils from groundnut, pongamia, and sunflower are often used for the preparation of liquid formulations. Jayasudha et al. (2018) confirmed the effectiveness of the oils on the viability of a consortium made up of 4 microorganisms for 3 months of storage. Wet solid formulations are obtained by mixing microbial biomass with solid supports, called carriers, that may consist of alginate, clay, peat and biochar (Loján et al., 2017; Riseh et al., 2021). This kind of formulations after their application ensures a gradual and controlled release of microorganisms into the rhizosphere (Liffourrena and Lucchesi, 2018; Naik et al., 2020). In a particular study conducted with microcapsules, obtained with alginate in combination with whey proteins, a slow release of *Pseudomonas fluorescens* VUPF506 cells that lasted for 60 days was observed. The authors of this research found that this behavior can be justified by the moisture content and swelling index of the microcapsules (Fathi et al., 2021).

In recent years, research has been published highlighting the role of biochar as a microbial vector which, thanks to its porosity and large surface area, creates a microenvironment suitable for the growth of microorganisms, protecting them from soil predators (Ajeng et al., 2020; Mącik et al., 2020; Shabir et al., 2024). Jabborova et al. (2020) evaluated the effect of different quantities of corn biochar (1 and 3%) in combination with different PGPRs (*P. putida* and *B. japonicum*) finding a high availability of nutrients in the soil. Studies on the shelf-life of the formulation made up of *Bradyrhizobium japonicum* (CB1809) and biochar revealed high survival with values of up to 95% viability after 90 days (Shabir et al., 2024). In liquid and solid wet formulations, during storage the microbial cells are exposed to a high-water content therefore refrigeration is required which complicates the marketing and distribution phase. However, this disadvantage can be compensated by missing of expansive drying processes.

Dry solid formulations, on the other hand, have a longer shelf life, up to 2 years, and microorganisms can survive at higher temperatures than wet formulations. These can be powders or granules and involve the use of organic, inorganic or synthetic carriers that are easy to process and sterilize (Romano et al., 2020). The drying process of microorganisms can be carried out by mild technologies, such as spray- and freeze-drying, or by air drying immobilization on solid supports such as talc, zeolite, bentonite, etc. Freeze drying can be considered one of the best techniques for drying microorganisms as it is able to guarantee high vitality at the end of the process; however, the diffusion of this technology is severely limited by its high cost. Spray-drying, on the other hand, is probably the most used method because it is rather cheap and simple (Arumugham et al., 2023). In many cases, arabic gum, trihalose, skim milk, or maltodextrin are added to the biomass to protect, by microencapsulation, microbial cells from the strong stress caused by the drying process (Kumar et al., 2022). The study conducted by Stojanović et al. (2022) aimed to obtain a new commercial biofertilizer by spray-drying with maltodextrin as a carrier. After storage at room temperature for a period of 1 year, the microbial formulation consisting of *B. subtilis* NCIM 2063 showed a high survival rate. In a work conducted by Sohaib et al. (2020) the agronomic effect on wheat of a formulation consisting of a microbial consortium mixed in zeolite was evaluated. The formulation was obtained by inoculating 1 kg of carrier with 100 mL of bacterial suspension (10^8^ cells mL^−1^). The authors conclude that zeolite is characterized by a high-water retention capacity and, when used in these ratios, allows the suspension to be dehydrated and stabilized. Therefore, zeolite creates a favorable environment in terms of humidity which allows microorganisms to continue their metabolic activity and reduces the risk of microbial contamination. All this leads to an increase in the shelf-life of the formulation. A significant increase in the shelf-life of a formulation based on *P. fluorescens* LBUM677 and *P. synxantha* LBUM223 was observed using a mixture of talc, carboxymethylcellulose (10% w/w) and calcium carbonate (Novinscak and Filion, 2020). The authors report that the viability of microorganisms, after a decrease recorded in the first 15 days, remains constant for 180 days.

**Figure 5:**
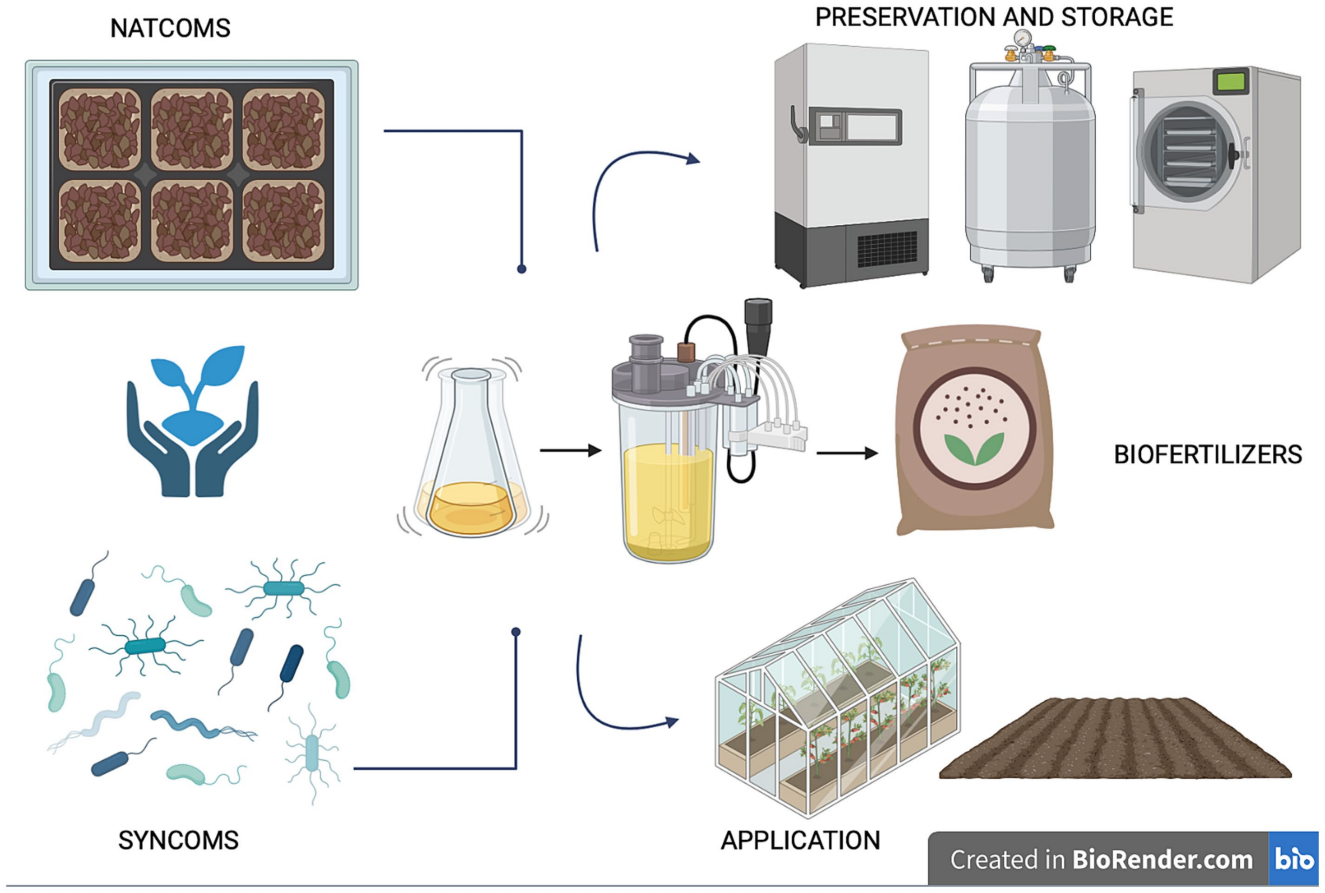
The microbiome-based approach: from selection to field application and storage.

In conclusion, the production of PGPR with the use of agro-industrial wastes as growth substrates, can represent a winning strategy in the perspective of circular bioeconomy because it lowers production costs, making materials otherwise destined for disposal profitable and presents a more sustainable environmental footprint. The results obtained from applications in the field of different formulations both liquid and solid are very promising and the type of formulation to be developed depends on different parameters such as effectiveness, stability, cost-effectiveness and ease of application (Figure 5).

## 9 Conclusion

In recent years, the extraordinary value of the microbiome for sustainable agriculture has been realized, and efforts are now being made to understand how to harness it to improve crop production and soil health. The goal of researchers is to define and apply microbiome-based solutions to have a healthy and useful microbiome for plants. The challenge is to identify, isolate and study “good” bacteria and then reproduce and apply them to crops. Starting from the soil microbiome investigation by omics strategies, efficient microbiome-based solutions could be produced. The use of soil microorganisms as soil fertilizers and plant strengtheners in synthetic or natural microbial consortia have become an ecologically favorable alternative to supplement inorganic inputs and promote plant development and health. Selecting and bringing beneficial microbiomes into the field may not be enough. Many researchers have addressed the issue of microbial inoculants and have tried to ensure and secure efficacy even in the open field, not always successfully. It has been seen how agricultural practices or plant genotype can influence the plant microbiome, and thus its functioning. Several avenues are therefore open to a new generation of inoculants and the application of microbiomes in agriculture that could initiate a new green revolution that is much more sustainable than the previous one. New efforts are necessary for translating the potential of microbiome-based solutions into products for farmers and agrifood companies. Industrial and academic partners are calling for public-private partnerships to favor the scaling-up of microbial productions and their commercialization translating the scientific knowledge into new products and treatments.

## Footnotes

^1^https://joey711.github.io/phyloseq/

^2^https://github.com/vegandevs/vegan

^3^https://simbaproject.eu

^4^https://www.sus-mirri.it/project/

^5^https://zenodo.org/records/10887823

^6^https://www.microbeproject.eu

^7^https://www.cabi.org/projects/the-european-microbiome-biobanking-ri-enabler/

^8^https://www.microbiomesupport.eu

^9^https://www.marketsandmarkets.com/Market-Reports/agricultural-inoculants-market-152735696.html
